# *rab-33* is not required for cuticle integrity in *Caenorhabditis elegans*

**DOI:** 10.17912/micropub.biology.001516

**Published:** 2025-03-16

**Authors:** Emily Williams, Nico Pinzon, Grace Semrau, Vicky Pete, Mary Elizabeth Gabrielle, Rachid El Bejjani

**Affiliations:** 1 Institute for Neurodegenerative Disease, Massachusetts General Hospital, Boston, Massachusetts, United States; 2 Biology, Davidson College, Davidson, North Carolina, United States

## Abstract

Rab GTPases are master regulators of intracellular transport. We previously showed that a *rab-6.2* deletion leads to a compromised cuticle, glycosylation defects, and increased axon regeneration. Mammalian orthologs of *rab-6.2* and *rab-33* have been shown to mediate Golgi trafficking of cargo in mammalian cells, including that of glycosyltransferase enzymes. We engineered a novel STOP-IN putative null allele of *rab-33 ( axr2 )* to determine if loss of function of *rab-33* phenocopies the phenotypes seen in *rab-6.2 ( ok2254 ).* Our results suggest that *rab-33* is not required for cuticle integrity.

**Figure 1. CRISPR Design and Cuticle Integrity Characterization of rab-33 mutants:**
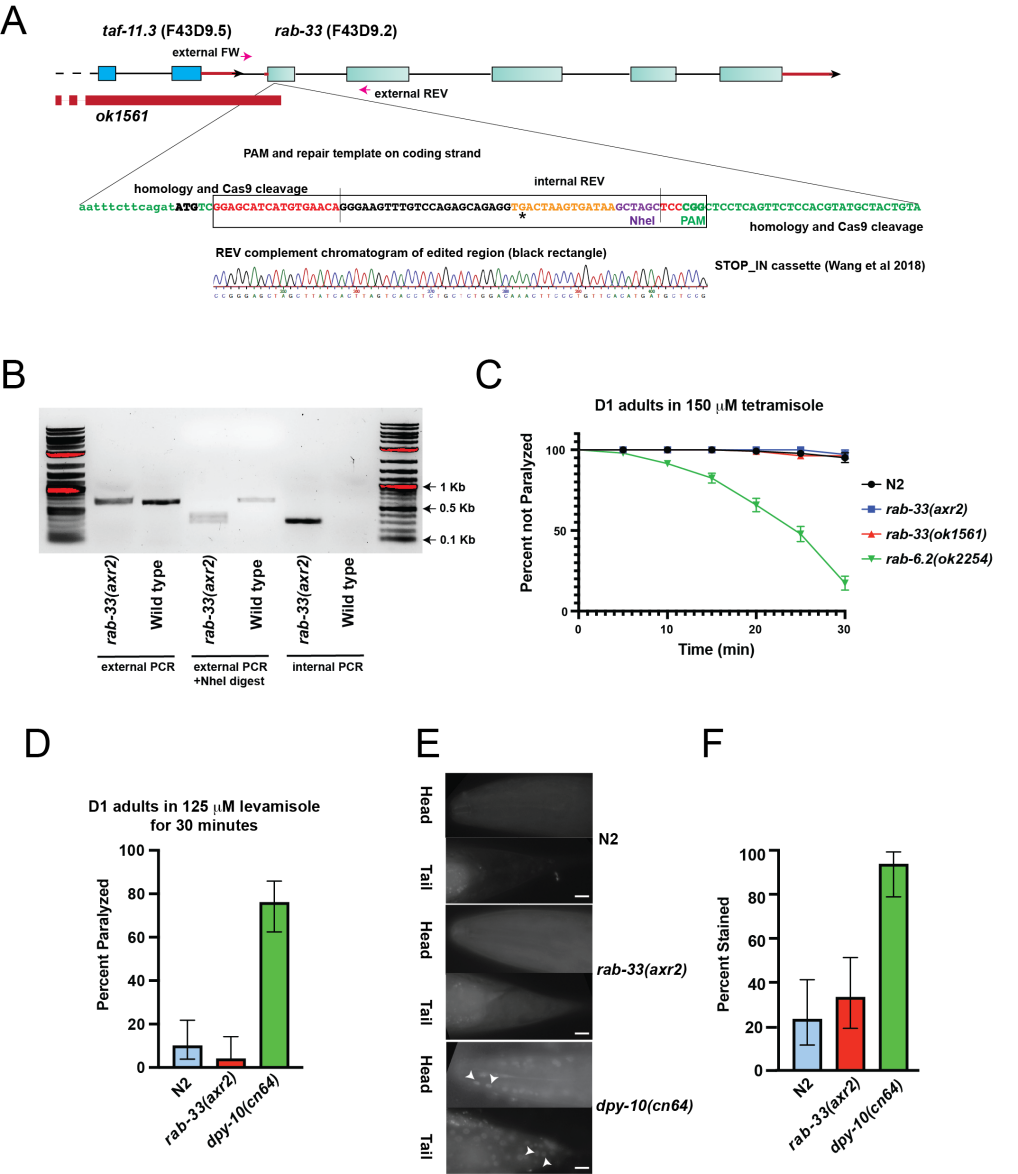
A) Diagram showing the *rab-33* and *taf-11 .3* loci and the *ok1561* deletion, dashed lines represent additional sequence upstream of the *rab-33* locus. The diagram also shows the genetic construct (STOP-IN cassette, adapted from Wang et al. 2018) used in this study and the CRISPR insertion site in the *rab-33* gene; HDR arms are highlighted (green) on both ends of the construct, Synthetic *Nhe-1* restriction enzyme site (purple), early stop codon is noted by an asterisk, external and internal genotyping primers are shown. Lower panel shows sanger sequencing data using a rev primer, confirming the correct insertion of the STOP-IN cassette in *rab-33 ( axr2 )* homozygotes. B) Genotyping results confirming correct insertion of STOP-IN cassette in our mutants. The external amplicon is also digested with NheI to confirm insertion. C) Results of 150 μM Tetramisole paralysis assays on day-1 adult N2 worms (wild type), *rab-6.2 ( ok2254 )* worms, *rab-33 taf11-.3 ( ok1561 )* and the newly engineered *rab-33 ( axr2 )* . P-value < 0.0001, Log-rank (Mantel-Cox) test. D) Results of 125 μM Levamisole paralysis assays after 30 minutes on day 1 adult N2 worms, *dpy-10 ( cn64 )* worms, and *rab-33 ( axr2 ).* Error bars denote standard deviation. P-value < 0.001. E) images of heads and tails of N2 , *dpy-10 ( cn64 )* , and *rab-33 ( axr2* ) D1 adult worms stained with Hoescht 33258. Arrowheads show stained nuclei in the *dpy-10 ( cn64 )* worms, which both the N2 and *rab-33 ( axr2 )* do not exhibit. F) percentage of animals displaying nuclear stains in heads or tails. Scale bar = 20mm. Error bars denote standard deviation. p < 0.001.

## Description

Rab proteins regulate intracellular transport by acting as molecular switches and binding a series of effector proteins that mediate the transport of various vesicles in eukaryotes (Martinez-Arroyo *et al.* 2021). The mammalian Golgi cisternal Rab proteins, including RAB6 and RAB33, are well conserved, and mutations in the human ortholog of *C. elegans rab-33* or in genes that interact with the *rab-6.2* ortholog are respectively associated with Smith-McCort Dysplasia and Gerodermia Osteodysplastica (Hennies *et al.* 2008; Dupuis *et al.* 2013). In HeLa cells, RAB6 and RAB33 coordinate some overlapping functions in Golgi trafficking, including the transport of glycosyltransferase enzymes suggesting a potentially shared mechanism of action in other organisms and tissues (Jiang and Storrie 2005; Starr *et al.* 2010). More recently, RAB6 and RAB33 have been associated with exocytosis and localized secretion (Fourriere *et al.* 2019; Homma *et al.* 2019; Bjornestad *et al.* 2022). Since the mammalian orthologs of *rab-6.2* and *rab-33* share some functions in Golgi transport and exocytosis, we set out to determine if *rab-33* mutant *C. elegans* have compromised cuticle integrity similarly to *rab-6.2* mutant worms.

We previously showed that *C. elegans* mutants *rab-6.2 ( ok2254 )* have compromised structural integrity and a permeable cuticle (Kim *et al.* 2019). *rab-6.2 ( ok2254 )* are also resistant to *M. Nematophilum* infection, a phenotype previously associated with defects in cuticle glycosylation (Gravato-Nobre *et al.* 2005; Kim *et al.* 2019). We hypothesized that this phenotype may be caused by defective intracellular trafficking of protein components of the cuticle or of glycosylation enzymes in *rab-6.2 ( ok2254 )* mutants. Because mammalian RAB6 and RAB33 have been shown to coordinate Golgi transport, including that of glycosylation enzymes, we hypothesized that, like *rab-6.2* , *C. elegans* *rab-33* may also be involved in maintaining cuticle integrity.

*ok1561* is the only allele of *rab-33* that is available at the *C. elegans* Genetics Center; however, it also deletes another gene, *taf-11 .3.* To investigate the role of *rab-33* specifically, we generated a novel *rab-33* mutant allele by inserting a STOP-IN cassette into the first exon using CRISPR/Cas9 editing as described by (Wang *et al.* 2018) ( Figure 1A ). We used a dominant mutation of *dpy-10 ( cn64 )* as a co-conversion marker to isolate *rab-33 ( axr2 )* (Arribere *et al.* 2014). The STOP-IN insertion in *axr2* results in an early stop codon, truncating all predicted functional domains of *rab-33* downstream of a 15 amino acid peptide ( Figure 1A ). We confirmed the correct integration of our construct by genotyping with a set of external primers that amplify a 43-nucleotide amplicon containing a synthetic NheI restriction site and with and internal PCR that only amplifies from genomic DNA templates containing the synthetic STOP-IN cassette ( Figure 1B ). Finally, correct insertion of the STOP-IN cassette was confirmed by Sanger sequencing (see supplementary materials).

We next tested the cuticle integrity of our *rab-33 ( axr2 )* mutants by assaying paralysis after exposure to exogenous paralytic agents. Because STOP-IN alleles may induce transcriptional adaptation, we also tested *rab-33 ( ok1561 )* which should avoid this response due to the deletion of the *rab-33* start codon and promoter ( Serobyan et al. 2020). We show that *rab-33 ( axr2 )* mutants paralyzed similarly to wild type ( N2 ) worms and to *rab-33 ( ok1561 )* but were significantly more resistant to tetramisole than *rab-6.2 ( ok2254 )* worms, known to be hypersensitive to exogenous chemicals because of previously reported cuticular defects ( Figure 1C ) (Kim *et al.* 2019). We obtained similar results when we repeated the experiment using Levamisole as a paralytic agent and mutants known to be hypersensitive, *dpy-10 ( cn64 )* as a positive control (Sandhu *et al.* 2021) ( Figure 1D ).

Tetramisole and Levamisole hypersensitivity can result from a defect in inhibitory neurotransmission and other synaptic defects or from an increase in permeability due to a compromised cuticle barrier (Jorgensen 2005; Rand 2007). To directly assess cuticle permeability, we exposed live, non-permeabilized worms to a DNA staining protocol. Under these staining conditions, the DNA stain Hoechst will only penetrate the cuticle and stain nuclei in mutant animals with demonstrated cuticular defects (Kage-Nakadai *et al.* 2010; Kim *et al.* 2019). After blindly scoring and quantifying our imaging results, we determined that the permeability of the cuticle of *rab-33 ( axr2 )* homozygote worms to Hoechst is indistinguishable from wild type and different from that of *dpy-10* worms which show increased permeability to Hoechst as previously described ( Figure 1 E and F).

## Methods

*Strain Maintenance*

*C. elegans* strains were grown and maintained on nematode growth media (NGM) plates seeded with *E. coli* OP50 and kept at 20°C. A list of strains generated during this work and used in the experiments is available in (Table 1). Some strains were provided by the CGC, which is funded by NIH Office of Research Infrastructure Programs (P40 OD010440).

*CRISPR-Cas9 Construct for rab-33 ( axr2 )*

A crRNA targeting the first exon of *rab-33* and a previously described *dpy-10* crRNA were respectively assembled into functional guide RNA by hybridizing with a synthetic tracrRNA as previously described (Wang *et al.* 2018). The pre-assembled guide was then mixed with purified spCas9 and two single stranded synthetic repair templates, one containing the STOP-IN cassette flanked by 35 nt homology on either side of the cut in the *rab-33* gene and another repair template containing the previously described *cn64* point mutation as a co-CRISPR marker (Arribere *et al.* 2014). Wild type N2 worms were injected with a mixture containing the CRISPR mix described above and a *myo-3p* :mCherry co-injection marker. F1 *roller* or *dpy* animals were picked and F2s were genotyped for the presence of the *rab-33* STOP_IN cassette using the internal primer and then external primers followed by NheI digest ( Figure 1A- B and Table 2). The new allele was then confirmed by Sanger Sequencing of the external PCR amplicon and outcrossed 2X. Synthetic RNA and DNA oligonucleotides, and the purified SpCas9 were purchased from IDT.

*Cuticle Permeability Assays*

Chemical paralysis assay plates were made by adding Tetramisole hydrochloride (Sigma-Aldrich) or Levamisole Hydrochloride (MP Biomedicals) to unseeded NGM plates at 150 μM and 125 μM concentrations, respectively. One-day-old adults of each strain were transferred onto 150 μM Tetramisole plates, and paralysis was blindly scored every 5 minutes for 30 minutes total. One-day-old adults of each strain were transferred onto 125 μM Levamisole plates, and paralysis was blindly scored once after 30 minutes. In both cases, paralysis was defined as the inability to move forward or backward when prodded three times consecutively with a platinum wire pick. One-day-old adults were incubated for 30 minutes in Hoechst 33258 Pentahydrate (Invitrogen, molecular probes) diluted to 1 μg/mL in M9, followed by washing three times with M9 buffer. These worms were mounted onto glass slides and immobilized using Levamisole and Tricaine (TCI Chemicals). Imaging was performed as previously reported (Kim *et al.* 2019). Images were blindly scored and quantified ( Figure 1F ).

## Reagents

**Table 1.** *C. elegans* strains used in this study.

**Table:** 

Strain	Genotype	Available from
N2	*C. elegans* wild isolate	CGC
REB115	*rab-33 ( axr2 ) *	This study
TN64	*dpy-10 ( cn64 ) *	CGC
RB1376	*rab-33 & taf-11 .3( ok1561 ) *	CGC

**Table 2.** Oligonucleotide sequences for CRISPR and genotyping.

**Table:** 

oligonucleotide name	Sequence
*rab-33* external FW	CACCGTATGGAGCGAAGTATTGAC
*rab-33* external REV	TGCTGATGGATGTGTTGGTTGG
*rab-33* internal REV	GCTTATCACTTAGTCACCTCTGCTC
*rab-33 ( axr2 )* crRNA	GGAGCATCATGTGAACATCC
*rab-33 STOP_IN repair ssODN *	AATTTCTTCAGATATGTCGGAGCATCATGTGAACAGGGAAGTTTGTCCAGA GCAGAGGTGACTAAGTGATAAGCTAGCTCCCGGCTCCTCAGTTCTCCACGTA TGCTACTGTA
*dpy-10* co-CRISPR crRNA and repair template	From (Arribere *et al.* 2014)

**Table 3.** Chemical reagents used in this study.

**Table:** 

Reagent	Available from
Tetramisole Hydrochloride	Sigma-Aldrich
Levamisole Hydrochloride	MP Biomedicals
Tricaine	TCI Chemicals
Hoechst 33258 Pentahydrate	Invitrogen, molecular probes

## Data Availability

Description: Sequence file for axr2 edit. Please download a plasmid editor to view the file.. Resource Type: Text. DOI: https://doi.org/10.22002/kb69a-3m263
